# Coorte retrospectiva de trissomia do cromossomo 18 (síndrome de Edwards) no sul do Brasil: Retrospective cohort of trisomy 18 (Edwards syndrome) in southern Brazil

**DOI:** 10.1590/1516-3180.2013.79900715

**Published:** 2014-11-07

**Authors:** Daniela Denardin, Fabíola Elizabete Savaris, André Campos da Cunha, Rosilene da Silveira Betat, Jorge Alberto Bianchi Telles, Luciano Vieira Targa, Aline Weiss, Paulo Ricardo Gazzola Zen, Rafael Fabiano Machado Rosa

**Affiliations:** I MD. Physician, Residency Program on Obstetrics and Gynecology, Hospital Materno Infantil Presidente Vargas (HMIPV), Porto Alegre, Rio Grande do Sul, Brazil.; II MD. Obstetrician, Fetal Medicine, Hospital Materno Infantil Presidente Vargas (HMIPV), Porto Alegre, Rio Grande do Sul, Brazil.; III MD. Fetologist, Fetal Medicine, Hospital Materno Infantil Presidente Vargas (HMIPV), Porto Alegre, Rio Grande do Sul, Brazil.; IV MD. Pediatric Radiologist, Hospital Materno Infantil Presidente Vargas (HMIPV), Porto Alegre, Rio Grande do Sul, Brazil.; V MD. Neonatologist, Hospital Materno Infantil Presidente Vargas (HMIPV), Porto Alegre, Rio Grande do Sul, Brazil.; VI PhD. Adjunct Professor of Clinical Genetics and of the Postgraduate Program on Pathology, Universidade Federal de Ciências da Saúde de Porto Alegre (UFCSPA), and Clinical Geneticist, Universidade Federal de Ciências da Saúde de Porto Alegre (UFCSPA) and Complexo Hospitalar Santa Casa de Porto Alegre (CHSCPA), Porto Alegre, Rio Grande do Sul, Brazil.; VII PhD. Clinical Geneticist, Universidade Federal de Ciências da Saúde de Porto Alegre (UFCSPA), Complexo Hospitalar Santa Casa de Porto Alegre (CHSCPA) and Hospital Materno Infantil Presidente Vargas (HMIPV), Porto Alegre, Rio Grande do Sul, Brazil.

**Keywords:** Trisomy, Chromosomes, human, pair 18, Karyotype, Prenatal diagnosis, Survival analysis, Trissomia, Cromossomos humanos par 18, Cariótipo, Diagnóstico pré-natal, Análise de sobrevida

## Abstract

**CONTEXT AND OBJECTIVE:**

Trisomy 18 (T18), or Edwards syndrome, is a chromosomal disease characterized by a broad clinical picture and a poor prognosis. Our aim was to describe clinical, radiological and survival data of a cohort of patients prenatally diagnosed with T18.

**DESIGN AND SETTING:**

Retrospective single cohort in the Fetal Medicine Service of Hospital Materno Infantil Presidente Vargas (HMIPV).

**METHODS:**

All sequential patients with T18 registered at the Fetal Medicine Service of HMIPV between January 2005 and September 2013 were considered. We gathered their clinical, radiological and survival data and used the Kaplan-Meier test for survival analysis.

**RESULTS:**

Ten patients were diagnosed with T18, of whom seven (70%) were female. The majority (90%) were referred due to malformations seen on ultrasound. The mean gestational age at the first evaluation was 25.5 weeks. At karyotyping, the defects were considered multiple in only four patients (40%). All the fetuses presented full trisomy of chromosome 18. The main abnormality observed was congenital heart disease (n = 7). Intrauterine death occurred in half of the patients (50%). All live patients (n = 5) were born through cesarean section presenting low weight and low Apgar scores. The median length of survival after birth was 18 days.

**CONCLUSIONS:**

T18 is associated with a high risk of fetal and neonatal death. The majority of the patients present major malformations identified through ultrasound, such as congenital heart defects, which could help in identifying such cases prenatally.

## RESUMO

**CONTEXTO E OBJETIVO:**

A trissomia do cromossomo 18 (T18), ou síndrome de Edwards, é uma doença cromossômica caracterizada por um quadro clínico amplo e prognóstico pobre. Nosso objetivo foi descrever os dados clínicos, radiológicos e de sobrevida de uma coorte de pacientes com diagnóstico pré-natal de T18.

**TIPO DE ESTUDO E LOCAL:**

Coorte única retrospectiva no Serviço de Medicina Fetal do Hospital Materno Infantil Presidente Vargas (HMIPV).

**MÉTODOS:**

Foram considerados todos os pacientes consecutivos com T18 registrados no Serviço de Medicina Fetal do HMIPV entre janeiro de 2005 e setembro de 2013. Foram coletados os seus dados clínicos, radiológicos e de sobrevida. Foi utilizado o teste de Kaplan-Meier para análise de sobrevida.

**RESULTADOS:**

10 pacientes foram diagnosticados com T18, 7 (70%) do sexo feminino. A maioria (90%) foi encaminhada devido a malformações detectadas no ultrassom. A média da idade gestacional na primeira avaliação foi de 25,5 semanas. Ao cariótipo, os defeitos foram considerados múltiplos em apenas 4 pacientes (40%). Todos apresentaram trissomia livre do cromossomo 18. A principal anormalidade observada foi a cardiopatia congênita (n = 7). Morte intraútero ocorreu em metade dos pacientes (50%). Todos os pacientes vivos (n = 5) nasceram através de parto cesáreo, apresentando baixo peso e baixos escores de Apgar. A mediana de sobrevida após o nascimento foi de 18 dias.

**CONCLUSÕES:**

A T18 associa-se a risco elevado de morte fetal e neonatal. A maioria dos pacientes apresenta malformações identificadas através do ultrassom, como cardiopatias congênitas, que poderiam auxiliar na sua identificação pré-natal.

## INTRODUCTION

Trisomy 18 (T18) was one of the first chromosomal abnormalities to be described. It was first reported by Edwards et al. in 1960 and, therefore, is also known as Edwards syndrome. Nowadays, it is considered to be the second most common chromosomal abnormality involving the autosomes, only behind trisomy 21 (Down syndrome).^1^^,^^2^ It has an estimated prevalence of approximately 1:3,600-8,500 live births.^3^ T18 is clinically characterized by a broad clinical picture, with more than 130 different findings already described, and a prognosis that is considered poor.^2^^,^^4^ Most fetuses diagnosed during gestation are spontaneously aborted and, among those that are born alive, most die within the first six months.^1^^,^^2^^,^^4^

In Brazil, prenatal identification of patients with T18 is important in determining issues relating to their evolution and prognosis, as well as their birth and clinical management. Many countries have also adopted a more interventionist stance and have made more investment in cases that come to birth, thereby increasingly respecting family autonomy in decision-making.^1^^,^^2^ However, in Brazil, there is a paucity of studies evaluating both the prenatal diagnosis and the natural history of patients with T18, especially from the beginning of pregnancy.^4^^,^^5^

## OBJECTIVE

Our aim was to describe clinical, radiological and survival data from a cohort of patients prenatally diagnosed with T18.

## METHODS

All sequential patients with T18 registered at the Fetal Medicine Service of Hospital Materno Infantil Presidente Vargas between January 2005 and September 2013 were considered. We gathered clinical, radiological and survival data on the patients from their medical records. This project was approved by the hospital’s Research Ethics Committee.

The information retrieved from medical records consisted of the reason for referral; gestational age at first assessment; maternal and paternal ages; maternal pregnancy history; presence of diseases and threatened abortion in the current pregnancy; results from first-trimester sonographic screening, echocardiography and karyotyping; delivery and perinatal features; and postnatal evaluation, survival and autopsy results.

Gestational age was determined according to the earlier ultrasound. The main reasons for fetal karyotyping were categorized as described by Kessler et al.^6^ The patients were classified according to the number of major and minor sonographic markers observed before and after puncturing for fetal karyotyping in accordance with Raniga et al.^7^ Abnormalities identified through imaging studies performed during prenatal care were also classified as single or multiple defects, in accordance with Staebler et al.,^8^ before and after performing fetal karyotyping. To determine the congenital heart defect observed, we used the classification suggested by Botto et al.^9^

The Kaplan-Meier test was used to construct the survival curve, by means of the BioEstat 5.0 software.

## RESULTS

Over this period of about nine years, ten patients were diagnosed with T18. The majority of them (90%) had been referred due to presence of malformations in an ultrasound evaluation (only one patient presented increased nuchal translucency). The mean gestational age at the first evaluation was 25.5 weeks. The maternal age ranged from 20 to 45 years (mean of 34.4 years) and the paternal age ranged from 30 to 40 years (mean of 33.9 years). Advanced maternal age (≥ 35 years) was observed in six cases (60%) (Table 1).

**Table 1.**
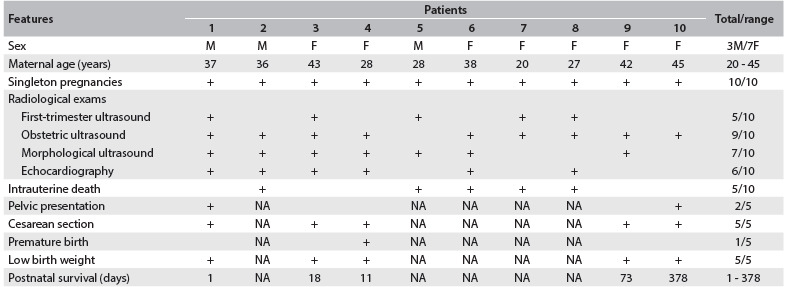
Gestational, perinatal and survival findings observed among the patients of the sample

M = male; F = female; NA = not applicable.

All the cases in the sample were singleton pregnancies. Regarding the maternal history of pregnancy, two mothers were primiparous (20%). One of them (10%) presented gestational diabetes. There were no cases of preeclampsia or even threatened abortion. Three mothers (30%) had family histories of malformations, and two of them had had malformed fetuses in a previous pregnancy. Half of the patients underwent first-trimester sonographic screening. Three of these screenings (60%) were considered normal. Two patients (40%) presented a cystic hygroma. In one of them, the nasal bone was not identified and there was tricuspid regurgitation. Abnormalities were seen in all cases in evaluations through obstetric and morphological ultrasound. Six patients (70%) underwent echocardiography and additional heart abnormalities were observed in three cases (all of these were ventricular septal defects) (Table 1 and 2).

**Table 2.**
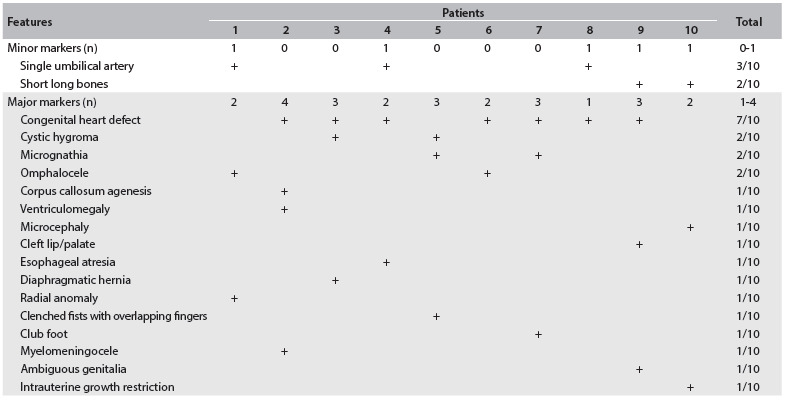
Prenatal findings among the patients of the sample by the end of the pregnancy, divided into minor and major markers as described by Raniga et al.^7^

**Figure 1.**
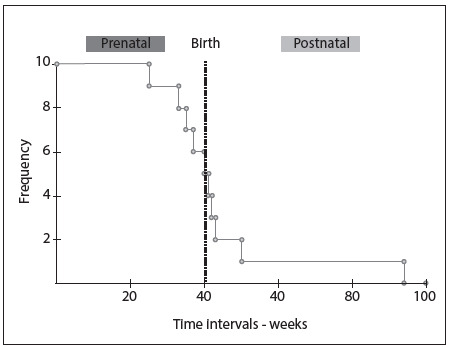
Kaplan-Meier curve showing the survival presented by the patients during the prenatal and postnatal periods. Note especially that half of them presented intrauterine death and that out of those who were born alive, three died within the first month of life and only one patient lived for longer than one year.

At the time of making the puncture for fetal karyotype analysis, the number of major markers was 1.8 per case and the number of minor markers was 0.3 per case. The defects were considered multiple in only four patients (40%). All the fetuses presented full trisomy of chromosome 18 in the karyotype analysis. By the end of the pregnancy, all the cases were characterized by multiple defects, i.e. in the six cases previously diagnosed as presenting isolated defects, additional major abnormalities were detected. The abnormalities observed can be seen in Table 2. According to Botto et al.,^9^ the main groups of heart malformations observed were septal (42.9%) and conotruncal defects (42.9%). The congenital heart defects observed in the present study consisted of ventricular septal defects (n = 3), double outlet of the right ventricle (n = 2), tetralogy of Fallot (n = 1) and hypoplastic left ventricle (n = 1). None of our cases tried to legally terminate the pregnancy. Intrauterine death occurred in half of the patients (50%) (Figure 1 and Table 1). By the end of the pregnancy, the number of major markers observed was 2.5 per case and the number of minor markers was 0.5 per case (Table 2).

Out of all the patients, 7 (70%) were female. All the live patients (n = 5) were born through cesarean section. Pelvic presentation was observed in two cases (40%). Only one was premature. Low birth weight (< 2,500 grams) was observed in all cases: the weight ranged from 1,460 to 2,475 grams, with a mean of 2,030 grams. The length ranged from 36 to 44.5 cm (mean of 41.3 cm) and the head circumference from 30 to 34.5 cm (mean of 32.7 cm) (Table 1). All patients (n = 5) presented Apgar score < 7 at first minute, and 3 at the fifth minute. Additional findings observed during the postnatal period and potentially identified through ultrasound during the prenatal period consisted of ambiguous genitalia (n = 2), clenched fists with overlapping fingers (n = 2) and micrognathia (n = 1). The survival of these patients ranged from 1 to 378 days (median of 18 days). Three patients (60%) died within the first month. Only one completed the first year of life. In none of the cases did the family authorize an autopsy (Figure 1 and Table 1).

## DISCUSSION

The Fetal Medicine Service of HMIPV is considered to be a reference in the state of Rio Grande do Sul for evaluation of pregnant women with fetuses suspected to have or diagnosed with some type of malformation, who have been attended within the Brazilian national health system (Sistema Único de Saúde, SUS). As seen from the results, the number of cases with prenatal diagnosis of T18 at this service is about one per year, which corresponds to about 10% of the demand from live births in the state (taking the year 2005 as the reference point, which had notifications of 11 cases). We believe that many cases of T18 were diagnosed only during the postnatal period. This is in accordance with the results obtained by Rosa et al. in the other study conducted in Brazil.^4^

There was a high rate of maternal age ≥ 35 years in our study, seen in 60% of the cases. The average maternal age observed (34.4 years) was similar to that described by Rosa et al.^4^ (33.9 years). The maternal age was similar to the paternal age (mean of 33.9 years). Diseases of pregnancy have been described among patients with T18.^4^ Rosa et al.^4^ observed a higher prevalence of preeclampsia. Sugayama et al.^5^ also described a high rate of unspecified hypertension in pregnancies of individuals with T18. A threat of abortion was also reported in 20% of the cases evaluated by Rosa et al.^4^ However, there were no cases of preeclampsia or threatened abortion in our sample. We cannot rule out the possibility that these findings may be related to our small sample size.

The first cases of prenatal diagnosis of T18 date from the 1970s.^2^ Today, diagnostic suspicion of T18 may be raised during the prenatal period through fetal ultrasonography, with measurement of nuchal translucency in the first trimester. This can be confirmed by fetal chromosome analysis through procedures such as puncturing the chorionic villi and amniocentesis.^2^ The finding of increased nuchal translucency is considered to be the most sensitive finding for diagnosing T18 up to the 16^th^ week of gestation.^10^ However, it was noteworthy in our sample that only one of the five patients (20%) who underwent this evaluation presented increased nuchal translucency. Moreover, this was the only case referred to our service due to this finding. This feature may have been related to our small sample size. On the other hand, it is interesting to note that only half of the sample was assessed by means of ultrasound during first trimester, thus showing that many patients are coming in late for evaluation.

Fetal echocardiography can also detect a congenital heart defect that may suggest the presence of T18. The sonographic finding of congenital heart disease is considered to be the most sensitive feature for diagnosing T18 after the 16^th^ week of pregnancy.^10^ In our sample, congenital heart disease was the main anomaly found during pregnancy (it was identified in 70% of the patients), and use of fetal echocardiography allowed detection of additional defects that had not been identified through morphological and obstetric ultrasound. This highlights the importance of performing echocardiography in these cases. The main defects, as observed in our sample, are of septal type, especially ventricular septal defects. Conotruncal defects, such as a double outlet of the right ventricle and tetralogy of Fallot, and left obstructive defects, such as hypoplastic left heart, are considered less common among patients with T18.^11^

Other common manifestations reported during pregnancy include intrauterine growth restriction and polyhydramnios. The latter has been described in 9-52% of pregnancies,^5^^,^^12^^,^^13^ and it seems related to abnormalities of sucking and swallowing presented by the fetus. However, despite its frequency, intrauterine growth restriction and polyhydramnios were uncommon in our sample. Polyhydramnios was observed only in the case with esophageal atresia (patient 4) (Table 1). We believe that in this case, the polyhydramnios was secondary to the digestive tract malformation presented by the fetus, which prevented adequate swallowing of amniotic fluid and hence led to accumulation around it.

It is important to highlight that the association between intrauterine growth restriction and major malformation consistent with the phenotype of T18 often leads to prenatally diagnosing it after the 20^th^ week of gestation. According to Viora et al.,^14^ modern ultrasound examinations clearly present high sensitivity (greater than 90%) for detecting fetuses with T18. This finding is also in agreement with the observations made by Yeo et al.,^15^ who found that multiple abnormalities were usually observed in fetal sonography, typically involving the brain, heart and upper limbs.^16^ In the study by Yeo et al.,^15^ all the fetuses had four or more abnormalities. In our sample, using the method of Raniga et al.,^7^ the number of major markers was 1.8 per case and the number of minor markers was 0.3 per case, at the time of karyotyping. Another important aspect of our sample was that at the time of fetal karyotyping, only four patients had multiple defects. Thus, it is important to have a high degree of suspicion in cases with malformations presenting a greater association with T18, such as omphalocele, diaphragmatic hernia, myelomeningocele and esophageal atresia.^1^^,^^2^

The most common chromosomal abnormality observed in these patients is full trisomy of chromosome 18,^1^^,^^2^ and all the patients of our sample presented this finding. Full trisomy of chromosome 18 has a relationship with advanced maternal age, due to the phenomenon of non-disjunction of chromosomes.^2^ In our sample, as pointed out earlier, we found a high rate of mothers aged over 35 years (60%). However, it is important to be aware that T18 can be secondary to other chromosomal abnormalities such as translocations, which may have important implications regarding genetic counseling for the patients and their families.^2^

We found that females predominated in our sample (70%). This finding is in accordance with the literature, which shows that the proportion of females has ranged from 56 to 78%.^4^^,^^5^^,^^17^^,^^18^^,^^19^ However, it is noteworthy that some authors have found equal frequencies of the sexes in evaluations performed during the prenatal period,^20^ especially before the 18^th^ week of gestation.^21^ These features may perhaps be related to the fact that female patients have been associated with a greater chance of being born alive and surviving for longer periods than boys.^2^

In our series, all the live patients were born through cesarean section. These high rates of cesarean section have also been frequently described in the literature (50 to 90%).^4^^,^^5^^,^^13^^,^^17^^,^^19^^,^^20^ It is noteworthy that there are some studies specifically drawing attention to this finding.^17^ In our sample, we believe that this feature was related to prenatal detection of major malformations, which thus influenced the choice of cesarean section as the delivery route.

Several studies have drawn attention to the high rate of prematurity described among patients with T18 (48%).^4^^,^^5^^,^^13^^,^^19^^,^^20^ However, in our series only one patient (20%) was premature. Regarding low birth weight, our frequency of 100% was similar to that described in the literature,^13^^,^^19^^,^^20^ including studies developed in Brazil.^4^^,^^5^ Regarding Apgar scores, the high rate of patients presenting scores below 7 (suggestive of some degree of anoxia) at the first and fifth minute that we observed in our study was similar to what was described by Lin et al.^19^ and Rosa et al.^4^

A significant proportion of the fetuses with T18 die while still in utero, as observed in our sample (50%). According to Morris and Savva,^22^ it is estimated that 72% of pregnancies with fetuses with T18 end in miscarriage or stillbirth between the 12^th^ week and full term. The median survival after birth among patients with T18 that has been reported in the literature has usually ranged from 2.5 to 14.5 days,^3^^,^^13^^,^^18^^,^^19^^,^^20^^,^^23^^,^^24^ and we obtained a similar value (18 days). It is noteworthy that the value described in the other study developed in Brazil, by Rosa et al.,^4^ was higher (31 days). Those authors associated this finding with possible postnatal selection, since most of the patients in their study had been referred by other medical units within the state and had not been born in the hospital. They did not rule out the possibility that patients with greater severity of disease may not have survived to the point of being referred to their hospital for evaluation and diagnosis. Interestingly, one of our patients (10%) presented survival longer than one year, and some other studies have reported that about 5-10% of the patients live longer this age.^1^^,^^2^ Some authors have also reported, as pointed out earlier, that female patients were more likely to be born alive and survive for a longer period of time than males.^3^^,^^19^^,^^24^ Moreover, the only patient who lived longer than one year was a female.

The birth of a child with T18 may represent a great challenge, with complex ethical implications. Even though termination of pregnancy in cases of fetuses with T18 is not permitted by Brazilian law, prenatal identification of such cases is of great importance to the family and the medical team, since it provides important information regarding prognosis and management for these patients. A multidisciplinary approach is usually necessary not only during pregnancy but also after birth. Moreover, diagnosing T18 is of critical importance for appropriate genetic counseling for families, so that correct risk calculation for future pregnancies can be made. Recurrence in cases of full trisomy of chromosome 18 is considered rare.

## CONCLUSIONS

T18 is a chromosomal disease associated with a high risk of fetal and neonatal death. The majority of the patients present major malformations identified through ultrasound, such as congenital heart defects, and this could help in prenatally identifying this condition. Among the live births, most have low birth weight and low Apgar scores. We believe that further studies, especially involving a larger number of individuals and different regions of the country, are required in order to better delineate the current setting of the prenatal diagnosis and natural history of patients with T18 in Brazil.
